# Supplementary material for: Procurement process and shortages of essential medicines in public health facilities: A qualitative study from Nepal
Source: PLOS Glob Public Health. 2024 May 2;4(5):e0003128. doi: 10.1371/journal.pgph.0003128 (PMC11065305; doi:10.1371/journal.pgph.0003128)
Supplement: S1 Text — (DOCX) [file pgph.0003128.s001.docx]

**Interview Guide**

**Medicine shortages, challenges with the procurement process, and inventory management system of essential medicines in Bagmati Province**

Greet and introduce yourself to the participant.

Welcome him/her to the interview and thank him/her for their willingness to meet with you and to participate in the study.

Elaborate on the aim of the study to the participant and obtain written consent from him/her to participate in the study before the interview commences.

Explain that the interview will be audio recorded for data collection, but assure participants that the information provided will remain confidential.

Reassure the participant to speak freely and honestly to get a clear and relevant perspective for the study

**Pose the following questions to the participant:**

**Demographic details**

Name of health facility:

Location:

Name of the managing staff:

Gender:

Age:

Qualification:

Designation:

No. of years of experience in current designation:

**Procurement of medicines (Only for province hospital, health office and municipality office)**

1. Brief on the routine procurement process in your health facility
2. How are the forecasting and quantification of essential medicines made?
3. How are essential medicines procured?
4. Which method is used for the procurement process?
5. Bidding
6. Direct purchase from the manufacturer
7. Purchase from wholesalers without bidding……
8. Received from the government channel
9. Others (specify)
10. Common issues experienced during the procurement.
11. How does the municipality/District/Province manage the budget for the essential medicines?
12. Please provide the budget for the essential medicines (Verify from the Budget and Program):
    1. Budget from the Federal Government:
    2. Budget from the Provincial Government:
    3. Budget from the Local Government:

**Inventory management system (All health facilities)**

1. Brief on your health facility's regular inventory management system.
2. Computerized inventory management system
3. Manual inventory management system
4. Problems with the computerized management system or manual inventory management system

Explore if there is insufficient storage space, Backup is not available, Need for continuous power supply, Extra staff requirement.

**Demand and Supply (Only to hospital, PHCs and HPs)**

1. In last 3 months, was there shortages of medicines in your health facility? If yes, name the type of medicine
2. What was your experience regarding the shortage of essential medicines last year?
3. At what time of the year does your health facility have a shortage of essential medicines?
4. What is the trend in the number of deficit essential medicines in the last year? (increased, decreased, similar, not clear)
5. What factors lead to a shortage of essential medicines in your health facility?
6. What measures are taken to tackle the shortage of essential medicines in your health facility?
7. Are there particular policies, guidelines or protocols regarding the essential medicines you follow to prevent their shortages in your health facility?
8. In the past, is there any instance that your health facility solved the problem of shortage of essential medicines? If yes, what was done?
9. What is the effect on you and your team due to the shortage of essential medicines? (Probe challenges from patients, frustration among staff)
10. What is the effect on patients due to the shortage of essential medicines? (Probe financial impact on trust with health facilities)
11. Which diseases are commonly seen in the patients visiting your health facility?
12. Which types of NCD-related medicines (diabetes, hypertension, and chronic kidney disease) are mostly consumed by your health facility?
13. Antibiotics
14. Antihypertensive
15. Antidiabetics
16. Others
17. Is the supply of the NCD medications affected as mentioned above? Why?

**Ask the questions below to all**

1. When do you demand the medicines/commodities to a higher level?
2. How do you calculate the demand quantity to be made from a higher level?
3. In the last year, did you get a regular supply of essential medicines from the higher level?
4. How did you receive the essential medicine from the higher level? Is there any mechanism for it?
5. What is the most common means of transportation from which you receive the medicine from the higher level?

**Question for District and Municipal Offices only:**

1. In the past year, did you not receive the supply of essential medicines as per the demand?
2. When are supplies of essential medicines and equipment done in health facilities?
3. Do you have a periodic supply plan? If Yes, confirm if there is any supply plan.
4. How is the quantity of supply of essential medicines determined for the health facilities?
5. How do you utilize LMIS report in decision-making for supply chain management of essential medicines?

**Opinion questions**

1. What were the common challenges in the procurement process faced during the COVID pandemic and other prior unfortunate situations? (Only for province hospitals, health offices, and local governments)
2. What is your opinion of what an effective procurement and inventory management system should be like?
3. Do you have any other suggestions for addressing the shortage of essential medicines?
4. Do you want to add anything?

Thank you
