# Supplementary material for: Procurement process and shortages of essential medicines in public health facilities: A qualitative study from Nepal
Source: PLOS Glob Public Health. 2024 May 2;4(5):e0003128. doi: 10.1371/journal.pgph.0003128 (PMC11065305; doi:10.1371/journal.pgph.0003128)
Supplement: Attachment — Submitted filename: Review of Shortage of Essential Medicines .docx [file pgph.0003128.s002.docx]

Shortage of Essential Medicines and Challenges with the Procurement Process in Public Health Facilities in a Low Resource Setting: A Qualitative Analysis from Nepal

Review

- I suggest revising the topic to something like "A qualitative research on Procurement and Shortages of Essential Medicines in Public Health Facilities in Low-Resource Settings in Nepal." By using the term "shortages of essential medicines," the reader is informed that there are existing challenges without explicitly stating the word "challenges."
- The authors failed to provide adequate background information for the study, which should have been included in both the introduction and the abstract.
- The authors claim that there are not many studies on this topic, but there are a lot of studies worldwide on stockouts, barriers, and supply chain issues related to essential medicine, perhaps not in Nepal.
- *However, interviews with health coordinators of the Hetauda sub-metropolitan city and Bagmati rural municipality could not be conducted*. The authors need to provide more explanation to the reader to justify this statement.
- The alignment of Line 205-Line 206 with the quote is incorrect. While it is true that there is nothing inherently wrong with using the same supplier repeatedly, the reasoning behind the health managers' decision to do so is not well-explained in these lines. The challenge they face is not effectively presented in this section.
- The quotes associated with Lines 261-263 cannot be interpreted as “participants DEMANDING a budget”.
- It was observed that some participants who used the Logistics Management Information System (LMIS) were unable to practice the First Expiry First Out (FEFO) method. Again the authors noted that not all participants had been trained in eLMIS. Thus, the authors needed to clarify which regions had LMIS and which ones did not have LMIS.
- In line 133, The authors have stated that two districts were excluded from the study because they lacked a rural municipality. It is unclear whether the interviews were only conducted in rural areas. Therefore, the authors need to provide information on their inclusion and exclusion criteria for selection.
- The authors included the names of the participants and work locations in the quotes. However, the wider audience may not be familiar with these names. It would be better to use classifications that are more understandable to the readers. Additionally, there is a risk that the anonymity of the participants may be compromised if they can be identified by their names and work locations.
- The author should incorporate more literature on barriers to essential medicine procurement. Many studies worldwide address this issue and could further enrich the discussion section.

The author must revise the manuscript before submission and in addition, take care of the following issues.

- To revise Line 246 which has a bracket.
- Line 420: The author should use the term borrowed not burrowed.
- Abstract: Remove the two full stops after the first line
- Line 89 the authors should use “Patients’ not patients”.
