# Supplementary material for: Procurement process and shortages of essential medicines in public health facilities: A qualitative study from Nepal
Source: PLOS Glob Public Health. 2024 May 2;4(5):e0003128. doi: 10.1371/journal.pgph.0003128 (PMC11065305; doi:10.1371/journal.pgph.0003128)
Supplement: Attachment — Submitted filename: Response to Reviewers.docx [file pgph.0003128.s003.docx]

Date: 16^th^ January 2024

Editor,

PLOS Global Public Health

Dear Editor,

I am writing to submit the revised version of my manuscript, titled **"Procurement process and shortages of essential medicines in public health facilities: A qualitative study from Nepal",** for consideration for publication in PLOS Global Public Health. I appreciate the constructive feedback the reviewers and the editorial team provided, and I believe that the revisions addressed the concerns raised.

In response to the reviewers' comments, I have carefully revised the manuscript, incorporating changes that enhance the work's clarity, rigor, and overall quality. To facilitate the review process, I have used the "Track Changes" feature in the document to highlight the modifications made. This should assist the reviewers and editorial team in identifying the revisions easily.

I have attached the revised manuscript and a detailed response to each reviewer's comments. I trust that these changes align with the standards and expectations of PLOS Global Public Health and contribute to the overall improvement of the manuscript.

I want to express my gratitude for the opportunity to submit this revised version. I am confident that the modifications strengthened the manuscript and aligned it more closely with the aims and scope of PLOS Global Public Health.

Thank you for your time and consideration. I look forward to the possibility of seeing my work published in PLOS Global Public Health and contributing to the scholarly discourse in our field.

Sincerely,

Sunil Shrestha ([sunilcresta@gmail.com](mailto:sunilcresta@gmail.com))

Reviewers' comments:

Reviewer's Responses to Questions

**Comments to the Author**

1. Does this manuscript meet PLOS Global Public Health’s [publication criteria](https://journals.plos.org/globalpublichealth/s/criteria-for-publication)? Is the manuscript technically sound, and do the data support the conclusions? The manuscript must describe methodologically and ethically rigorous research with conclusions that are appropriately drawn based on the data presented.

Reviewer #1: Yes

Reviewer #2: Partly

Reviewer #3: Yes

**Response to reviewers:** We have carefully addressed Reviewers comments, enhancing the methodological and ethical rigor. We welcome further suggestions to ensure the highest standards for publication are met.

2. Has the statistical analysis been performed appropriately and rigorously?

Reviewer #1: N/A

Reviewer #2: No

Reviewer #3: Yes

**Response to reviewers:** We have carefully reviewed the methods of analyzing qualitative data, ensuring accuracy and rigor in our analysis. Any identified issues were addressed in the revised manuscript to meet the highest standards of validity. We thank the reviewers for their valuable input.

3. Have the authors made all data underlying the findings in their manuscript fully available (please refer to the Data Availability Statement at the start of the manuscript PDF file)?

The [PLOS Data policy](https://journals.plos.org/globalpublichealth/s/data-availability) requires authors to make all data underlying the findings described in their manuscript fully available without restriction, with rare exception. The data should be provided as part of the manuscript or its supporting information, or deposited to a public repository. For example, in addition to summary statistics, the data points behind means, medians and variance measures should be available. If there are restrictions on publicly sharing data—e.g. participant privacy or use of data from a third party—those must be specified.

Reviewer #1: Yes

Reviewer #2: No

Reviewer #3: Yes

**Response to reviewers:** All the data underlying the findings are available within the manuscript itself.

4. Is the manuscript presented in an intelligible fashion and written in standard English?

PLOS Global Public Health does not copyedit accepted manuscripts, so the language in submitted articles must be clear, correct, and unambiguous. Any typographical or grammatical errors should be corrected at revision, so please note any specific errors here.

Reviewer #1: Yes

Reviewer #2: No

Reviewer #3: Yes

**Response to reviewers:** We appreciate the feedback from the reviewers regarding the clarity and language of the manuscript. We have thoroughly revised the manuscript, addressing typographical or grammatical errors to enhance clarity and language quality.

5. Review Comments to the Author

Reviewer #1: Article acceptable once all quantitative language is corrected in particular use of questionnaire should be replaced with interview guide.

**Response to reviewers:** We have replaced the questionnaire with an interview guide **[Addressed in Line 52]**.

Reviewer #2: The title mentioned " Shortage of essential medicines", kindly provide necessary data justifying your view point as it currently lacking in your manuscript.
**Response to reviewers:** Regarding the title critique from the reviewer, we acknowledge the importance of aligning the title with the presented data. Our study, focused on essential medicine shortages in public health facilities in low-resource settings in Nepal, extensively details the barriers contributing to these shortages. Specifically, we have addressed delays in procurement, inadequate storage space, lack of training on LMIS, insufficient IT resources, unrealistic demand estimation, transportation budget issues, and reliance on manual inventory management systems. By explicitly referencing these findings in the introduction, we aim to strengthen the connection between the title and the substantive data presented in the manuscript.

We appreciate the thoughtful comments from both reviewers, which have undoubtedly contributed to our research's refinement and overall quality. These revisions ensure that our discussion is more comprehensive, well-supported, and aligned with the study's title and objectives.

The enrollment of health facilities is not very convoluted. kindly make it simpler also include data such as number of each types of health facilities (PHC, CHC, Hospital etc.) available in each district, number of each types of health facilities selected from each district and method of selection of health facilities.
**Response to reviewers:** Thank you for your feedback. We selected one hospital, one health office, two primary health centers (PHCs) and four health posts from each of the districts. In addition, we selected two municipal health coordinators from each of the districts and a logistics personnel from the province. This resulted in 61 interviews, out of which 59 were completed. The sampling strategy for districts and municipalities is mentioned in the manuscript. We decided to select one PHC and two health posts from each of the selected municipalities. As our sampling strategy was to select two municipalities from each of the districts, this resulted in selecting two PHCs and four health posts from a district. Usually, a municipality has only one PHC. If there was no PHC in the municipality, we selected another health post from the same municipality. In a municipality, the number of health posts varies from 1 to 5 depending on the size and the number of former village development committees (VDCs) merged to form a municipality during federal restructuring. l

Out of total 59 interviews conducted, please elaborate the designation of interviewees. Present data in terms of percentage
**Response to reviewers:** We have now included the designation of interviewees and their distribution in percentage in Table 2 under the results section. We have categorized the study participants into (i) health post in-charge, (ii) PHC in-charge, (iii) health coordinators, (iv) store in-charge, hospital, (v)store in-charge, health office, (vi) chief, health office, (vii) other municipal staff, and (viii) logistics officer, HLMC.

How informed consent was taken ( via phone , google form etc.) ?

**Response to reviewers:** Thank you for your comments. This is the change, “*Due to COVID-19 pandemic, the predominant method for obtaining informed consent was either through verbal consent or email, utilizing digital signatures for authentication. Additionally, a limited number of consents were gathered in-person, ensuring strict adherence to COVID-19 protocols at that particular time.”* **[Addressed in Line 245-248]**

Results are not written clearly. number of questions under each theme and response for each question by different interviewees should be discussed in results.

**Response to reviewers:** Thank you for your comments. Since this study was conducted using inductive analysis, we did not have pre-determined themes. Instead, the findings from the interview were used to identify codes grouped into themes and later categorized under broad domain. We have discussed the responses of the interviewees under the suitable sub-themes. The interview guide is attached in the supplementary file. **[Addressed in Results section]**.

.

Reviewer #3: The article is good but requires some improvement and clarifications based on review I have attached in here including the background, and the discussion sections which requires more revision. This is a qualitative research and therefore did not require statistical analysis.

**Response to reviewers:** Thank you for the comments. We have reviewed the attached comments and tried our best to address this. We have now revised the background and discussion section.

Shortage of Essential Medicines and Challenges with the Procurement Process in Public Health Facilities in a Low Resource Setting: A Qualitative Analysis from Nepal

Review

- I suggest revising the topic to something like "A qualitative research on Procurement and Shortages of Essential Medicines in Public Health Facilities in Low-Resource Settings in Nepal." By using the term "shortages of essential medicines," the reader is informed that there are existing challenges without explicitly stating the word "challenges."

**Response to reviewers:** We appreciate the valuable suggestion from the reviewer. In response, we have revised the topic: **Procurement Process and Shortages of Essential Medicines in Public Health Facilities: A Qualitative Study from Nepal.' [Addressed in Lines 1-2 and 100-101]**

- The authors failed to provide adequate background information for the study, which should have been included in both the introduction and the abstract.

**Response to reviewers:** We appreciate the reviewer's feedback and recognize the importance of providing adequate background information for the study. In response to this comment, we have carefully reviewed and revised the introduction and the abstract to incorporate more detailed background information. We believe these revisions enhance the overall quality of the manuscript by addressing the highlighted concern. **[Addressed in Introduction Section]**

- The authors claim that there are not many studies on this topic, but there are a lot of studies worldwide on stockouts, barriers, and supply chain issues related to essential medicine, perhaps not in Nepal.

**Response to reviewers:** We appreciate the reviewer's feedback on the abundance of global studies on stockouts, barriers, and supply chain issues related to essential medicine—our claim in the manuscript aimed to highlight the relatively limited focus on Nepal within this literature. We have revised the relevant sections in both the introduction and the abstract to emphasize the need for context-specific insights into Nepal's health system. We hope this clarifies our perspective and addresses the concern raised. We believe the revised manuscript now accurately reflects the global context while underscoring the specific research gap in Nepal **[Addressed in Introduction Section]**.

- *However, interviews with health coordinators of the Hetauda sub-metropolitan city and Bagmati rural municipality could not be conducted*. The authors need to provide more explanation to the reader to justify this statement.

**Response to reviewers:** Health coordinators from Hetauda sub-metropolitan city and Bagmati rural municipality were not available for the interview. The reason was that one of the officials opted for in-person interview which was not possible due to movement required during COVID-19 while the other could not be approached due to poor mobile network communication despite multiple contacts. We have now included this description in the methods section. **[Addressed in Line No 199-204]**

- The alignment of Line 205-Line 206 with the quote is incorrect. While it is true that there is nothing inherently wrong with using the same supplier repeatedly, the reasoning behind the health managers' decision to do so is not well-explained in these lines. The challenge they face is not effectively presented in this section.

**Response to reviewers:** In Line 205-Line 206, the quote from the Logistic Officer, HLMC, Bagmati Province, is now better aligned to emphasize the challenges faced by health managers in the procurement process. The officer highlights, "Procurement process is time-consuming. So, we plan to initiate procurement in the first quadrimester and distribute it in the second." At the hospital level, the difficulties in the process are further explained by the Store In-charge from Province Hospital, Hetauda, who states, "It’s a difficult process, we have to arrange different lists, and the process is long. We cannot reach all the suppliers for the rate and quotation, so we have to ask suppliers in our roster for the drugs via phone. Medicine might get out of stock at any time." This adjustment aims to provide a clearer connection between the presented quote and the challenges inherent in the procurement process. **[Addressed in Line No 279-286]**

- The quotes associated with Lines 261-263 cannot be interpreted as “participants DEMANDING a budget”.

**Response to reviewers:** While reviewing the quotes associated with Lines 261-263, it is evident that the participants are not explicitly demanding a budget. For instance, the statement from the health office in Lalitpur emphasizes the need for the health office to have authority in procurement and strengthen its role in ensuring an adequate supply of essential medicines and other supplies in health facilities. Although a few participants at the health post level expressed a need for budget allocation to address the immediate shortage of medicines, it is essential to clarify that the overall tone of the quotes does not explicitly demand a budget but rather highlights the necessity for enhanced procurement authority and strengthened roles. We have now revised the sentence, “*A few participants at the health post level emphasized the need for budget allocation to address the immediate shortage of medicines..”* **[Addressed in Line No 344-345]**

- It was observed that some participants who used the Logistics Management Information System (LMIS) were unable to practice the First Expiry First Out (FEFO) method. Again the authors noted that not all participants had been trained in eLMIS. Thus, the authors needed to clarify which regions had LMIS and which ones did not have LMIS.

**Response to reviewers:** Thank you for bringing this to our attention. We appreciate the opportunity to clarify the eLMIS training status at our study sites. During the data collection, eLMIS training was specifically conducted at the HLMC and Municipality levels. However, it's important to note that this training did not extend to the health facilities within the municipality such as health posts and primary health centres.

Before the introduction of eLMIS training, a paper-based reporting system called LMIS was implemented across all hospitals and health facilities. This dual system transition likely contributed to the observed variations in applying the First Expiry First Out (FEFO) method.

- In line 133, The authors have stated that two districts were excluded from the study because they lacked a rural municipality. It is unclear whether the interviews were only conducted in rural areas. Therefore, the authors need to provide information on their inclusion and exclusion criteria for selection.

**Response to reviewers:** Thank you for your insightful comments. This is the changes “*We randomly selected six out of 13 districts in the Bagmati province, namely Chitwan, Nuwakot, Lalitpur, Sindhupalchowk, Makwanpur and Sindhuli. Kathmandu and Bhaktapur districts were excluded from the sampling frame as they lacked rural municipalities. Exclusion criteria were applied to districts lacking a rural municipality, emphasizing the intentional inclusion of districts with both municipality and rural municipality setups. This strategic approach assures a broader representation of diverse settings in our study sites, supporting our objective of comprehensively understanding the investigated factors.*.” **[Addressed in 173-182]**

- The authors included the names of the participants and work locations in the quotes. However, the wider audience may not be familiar with these names. It would be better to use classifications that are more understandable to the readers. Additionally, there is a risk that the anonymity of the participants may be compromised if they can be identified by their names and work locations.

**Response to reviewers:** We have incorporated the participants' work locations and designations in the manuscript, considering their relevance to the context. However, we acknowledge the reviewer's point about potential unfamiliarity with specific names and locations among the broader audience. To strike a balance, we have now used more general classifications that maintain anonymity while still indicating the participants' locations in a broader sense.

- The author should incorporate more literature on barriers to essential medicine procurement. Many studies worldwide address this issue and could further enrich the discussion section.

**Response to reviewers: Thank you for your observation.** We now have added more literature on barriers to essential medicine procurement for comparability. **[Addressed in Discussion section, Line 648-657]**

The author must revise the manuscript before submission and in addition, take care of the following issues.

- To revise Line 246 which has a bracket.
- Line 420: The author should use the term borrowed not burrowed.
- Abstract: Remove the two full stops after the first line
- Line 89 the authors should use “Patients’ not patients”.

**Response to reviewers:** We thank the reviewer for highlighting the manuscript's typographical error. We have incorporated revisions following the provided suggestions.
